# Hyper-Enhanced Production of Foreign Recombinant Protein by Fusion with the Partial Polyhedrin of Nucleopolyhedrovirus

**DOI:** 10.1371/journal.pone.0060835

**Published:** 2013-04-09

**Authors:** Sung Min Bae, Hee Jung Kim, Jun Beom Lee, Jae Bang Choi, Tae Young Shin, Hyun Na Koo, Jae Young Choi, Kwang Sik Lee, Yeon Ho Je, Byung Rae Jin, Sung Sik Yoo, Soo Dong Woo

**Affiliations:** 1 Department of Agricultural Biology, College of Agriculture, Life and Environment Sciences, Chungbuk National University, Cheongju, Republic of Korea; 2 Department of Agricultural Biotechnology, College of Agriculture and Life Science, Seoul National University, Seoul, Republic of Korea; 3 College of Natural Resources and Life Science, Dong-A University, Busan, Republic of Korea; 4 Research Institute for Agriculture and Life Sciences, Seoul National University, Seoul, Republic of Korea; 5 Choong-Ang Vaccine Laboratory, Daejeon, Republic of Korea; University of Florida, United States of America

## Abstract

To enhance the production efficiency of foreign protein in baculovirus expression systems, the effects of polyhedrin fragments were investigated by fusion expressing them with the enhanced green fluorescent protein (EGFP). Recombinant viruses were generated to express EGFP fused with polyhedrin fragments based on the previously reported minimal region for self-assembly and the KRKK nuclear localization signal (NLS). Fusion expressions with polyhedrin amino acids 19 to 110 and 32 to 110 lead to localization of recombinant protein into the nucleus and mediate its assembly. The marked increase of EGFP by these fusion expressions was confirmed through protein and fluorescence intensity analyses. The importance of nuclear localization for enhanced production was shown by the mutation of the NLS within the fused polyhedrin fragment. In addition, when the polyhedrin fragment fused with EGFP was not localized in the nucleus, some fragments increased the production of protein. Among these fragments, some degradation of only the fused polyhedrin was observed in the fusion of amino acids 19 to 85 and 32 to 85. The fusion of amino acids 32 to 85 may be more useful for the enhanced and intact production of recombinant protein. The production of E2 protein, which is a major antigen of classical swine fever virus, was dramatically increased by fusion expression with polyhedrin amino acids 19 to 110, and its preliminary immunogenicity was verified using experimental guinea pigs. This study suggests a new option for higher expression of useful foreign recombinant protein by using the partial polyhedrin in baculovirus.

## Introduction

The baculovirus expression vector system (BEVS) is an effective and widely used method for the production of recombinant proteins in insect cells or larvae. The most useful feature of BEVS is its ability to produce a particular protein in a cellular environment that supports post-translational modifications [1], [2]. Recently, many of the developments approved for use in animal and human drugs, such as several vaccines for porcine circovirus [3], human papillomavirus [4], cervical cancer [5] and influenza [6], [7], have accelerated the use of BEVS and increased its importance in the field [8]. Unlike other various expression systems, the development of BEVS is based on the strong promoter of polyhedrin [9], [10]. However, the expression efficiency of foreign proteins using the polyhedrin promoter could not obtain the protein yields observed for native polyhedrin. As a result of ongoing studies and efforts over the last decade, BEVS has evolved to overcome some of these technical issues [11], [12]. Many researchers have performed studies to resolve this limitation, including the alteration of promoter sequences, fusion expression with partial polyhedrin or various tagging signals and co-expression with regulatory proteins[13]–[18]. Although these techniques could enhance the expression efficiency somewhat, they were not entirely satisfactory.

Among these, we noted that fusion expression of the target protein with polyhedrin was most feasible because there have been many advanced reports describing the characteristics of the polyhedrin structure, assembly and localization since those prior studies [19], [20]. The polyhedrin amino acid sequence contains the KRKK sequence at positions 32–35 and functions as a minimal nuclear localization signal (NLS); additionally, the 19–110 region of polyhedrin is required to form supramolecular self-assembly into a nuclear occlusion-like particle [19]. We hypothesize that localization in the nucleus and assembly of recombinant proteins are very important factors related to higher levels of protein production, as they enhance the stability of the produced proteins. In particular, DNA viruses such as baculovirus inhibit the nuclear-cytoplasmic protein transport of the host cells to replicate the virus particle, preventing an antiviral response within the nucleus [21]. This suggests that the nuclear environment is better than the cytoplasm for stabilizing foreign protein. Because neither the influence of nuclear assembly nor the localization regions for the production of recombinant protein fusions expressed with polyhedrin has yet been evaluated, we investigated their influences on the production of recombinant protein.

In this study, we constructed a number of recombinant *Autographa californica* multicapsid nucleopolyhedroviruses (AcMNPVs) to express the fusion form of enhanced green fluorescence protein (EGFP) with several domains between amino acids 19 and 110 of polyhedrin. The results showed that all of the tested partial polyhedrin fusions increased the expression of EGFP, especially the fusion with amino acids 19–110 and 32–110 that localized the EGFP fusion in the nucleus; this fusion exhibited the highest expression and yielded protein levels similar to the levels observed with polyhedrin. In addition, we could achieve hyper-expression of the E2 protein of classical swine fever virus (CSFV) through a fusion with partial polyhedrin. Our results showed the optimal fusion expression region of polyhedrin for the hyper-expression of foreign protein, which resulted in a greater than 10 fold higher expression yield.

## Materials and Methods

### Cells and Viruses

The *Spodoptera frugiperda* continuous cell line IPLBSF-21(Sf21 cells) was maintained at 27°C in SF900 II serum-free medium (Gibco). The AcMNPV C6 and CSFV LOM strains (GenBank accession number EU789580) were used in this study. Routine cell culture maintenance and virus production procedures were performed according to the published procedure [2].

### Construction of Transfer Vector

The EGFP gene amplified from pEGFP (Clontech) with primer set EGFP-F/R (Table 1) was cloned in-frame into pBacPAK9 (Clontech) between the *Eco*R I and *Pst* I sites to generate pB9-EGFP for recombinant virus rAc-EGFP. The nucleotide sequence encoding polyhedrin amino acids 19 to 110 was amplified from genomic DNA of AcMNPV by using the PCR primer set Polh1 19-110-F/R and then cloned into the pB9-EGFP, resulting in pB9-19-110-EGFP for rAc19-110-EGFP. A NLS mutation fragment (^32^KRKK^35^ mutated to ^32^NGNN^35^) expressing amino acid residues 19 to 110 was amplified from pB9-19-110-EGFP by using the PCR primers NGNN19-110-F and EGFP-R. The NLS mutation fragment was also cloned into pBacPAK9, resulting in pB9-NGNN19-110-EGFP for rAcNGNN19-110-EGFP. Polyhedrin 32 to 110 coding region with EGFP was amplified from pB9-19-110-EGFP using the PCR primers Polh 32-F and EGFP-R and cloning into pBacPAK9, resulting in pB9-32-110-EGFP for rAc32-110-EGFP. Specific deletions within polyhedrin fragments 19–85, 32–85 and 32–81 were generated from pB9-19-110-EGFP by PCR and Splicing by Overlap Extension (SOE) as described previously [22]. Briefly, two nested primers were designed to be complementary and carried two segments corresponding to flanking sequences upstream and downstream of the region to be mutated. The sequences of the nested primers used in the PCR reactions utilized the PCR primer sets Polh 85-R (P2), Polh 85-F (P3) and Polh 81-R (P2), Polh 81-F (P3). Each of the primers was used in reactions with upstream or downstream outside primers Polh1 19-110-F (P1), Polh 32-F (P1) or EGFP-R (P4). The products were then mixed, denatured and used as the template in a third PCR amplification using only the outside primers (P1, P4). The final PCR products were cloned into the intermediate plasmid, and then each partial polyhedrin with the EGFP fragment was cloned into pBacPAK9, resulting in pB9-19-85-EGFP, pB9-32-85-EGFP and pB9-32-81-EGFP for rAc19-85-EGFP, rAc32-85-EGFP and rAc32-81-EGFP, respectively. The gE2 gene from nucleotides 2,441-3,439 of the CSFV LOM strain was generated by RT-PCR with primers CSFV E2-F and CSFV E2-TMR-R. The gE2 gene was synthesized without its transmembrane region (TMR) [23]. The plasmids were digested with *Eco*R I and *Pst* I and then cloned into pBacPAK9 to prepare pB9-E2-ΔTMR. The polyhedrin 19 to 110 coding region was amplified using the PCR primer set Polh2 19-110-F/R, and then it was cloned into pB9-E2-ΔTMR, resulting in pB9-19-110- E2-ΔTMR for rAc-19-110- E2-ΔTMR. Specific partial deletions of polyhedrin fragment 32-85 were generated from pB9-19-110- E2-ΔTMR by SOE. A PCR product (32-85-E2- ΔTMR) was amplified using the primers Polh 32-F (P1), CSFV 85-R (P2), CSFV 85-F (P3) and CSFV E2-TMR-R (P4) and then cloned into pBacPAK9 for rAc-32-85- E2-ΔTMR.

**Table 1:** Primers used for the amplification and sequencing in this study.

Name of primer^a^	Primer sequence^b^
**Polh1 19-110-F (P1)**	GAATTC*ATA*ATGAAGTACTACAAAAATTTAGGTG
**Polh1 19-110-R**	GAGCTC**CCATGG**TAACAATGGGGAAGCTGTCTTC
**Polh2 19-110-F (P1)**	**AGATCT** *ATA*ATGAAGTACTACAAAAATTTAGGTG
**Polh2 19-110-R**	**GAATTC** AACAATGGGGAAGCTGTCTTC
**NGNN19-110-F**	**AGATCT** ATAATGAAGTACTACAAAAATTTAGGTGCCGTTATCAAGAACGCTAATGGCAATAATCACTTC
**Polh 85-R (P2)**	GCTCACCATGGTAAGCTTCATCGTGTCGG
**Polh 85-F (P3)**	ACGATGAAGCTTACCATGGTGAGCAAGGG
**Polh 32-F (P1)**	**GAATTC** *ATA*ATGAAGCGCAAGAAGCACTTC
**Polh 81-R (P2)**	CCCTTGCTCACCATGTCGGGTTTAACATT
**Polh 81-F (P3)**	AATGTTAAACCCGACATGGTGAGCAAGGG
**EGFP-F**	**GAATTC** *GCCA* ***CC*** **ATGG**TGAGCAAGGGCGAGG
**EGFP-R (P4)**	**CTGCAG** TTACTTGTACAGCTCGTCCATGCC
**CSFV E2-F**	**GAATTC** *ATA*ATGCGGCTAGCCTGCAAGGAAGATT
**CSFV E2-TMR-R (P4)**	**CTGCAG** TCAGTCAGTCACGTCCAGG
**CSFV 85-R (P2)**	CTTGCAGGCTAGCCGCATAAGCTTCATCGTGTCGG
**CSFV 85-F (P3)**	ACGATGAAGCTTATGCGGCTAGCCTGCAAG

a For each truncation construct, forward primer (F) is in the forward orientation relative to the coding strand, and reverse primer (R) is in the reverse orientation relative to the coding strand.

b Restriction enzyme site shown in bold; Kozak consensus translation initiation sequence shown in italics.

### Generation of Recombinant Baculoviruses

Recombinant AcMNPVs expressing various encoded fragments under the control of the polyhedrin promoter were generated by co-transfection with each transfer plasmid and a defective viral genome, bAcGOZA DNA [24]. Transfection was performed using Cellfectin II™ (Invitrogen) reagent according to the manufacturer’s instructions, and the recombinant viruses were purified and propagated in Sf21 cells as described previously [25].

### Confocal Laser Scanning Microscopy

Sf21 cells were cultured on sterile cover slips (placed in six-well plates) and infected with virus at 5 MOI (multiplicity of infection). After 3 days infection, the cells were fixed by methanol for 3 min at −20°C, rinsed with PBS, and blocked with 2% bovine serum albumin for 30 min at 37°C. The nuclei were stained with PBS containing 10 µg/ml of propidium iodide (Invitrogen), and the cells were washed thoroughly. Cover slips were mounted on glass slides with one drop of 50% glycerol in PBS and air dried for 15 min. Visualization and localization of the nucleus and recombinant protein were conducted using a confocal laser scanning microscope (LSM 510, Zeiss) at 63X magnification and 8-bit resolution.

### Biochemical Fractionation

Sf21 cells were seeded at 1×10^6^ cells per well in six-well plates and infected with virus at 5 MOI. Cells were collected 72 h post-infection, washed with ice-cold PBS, and lysed by suspension in 500 ul of ice-cold TBN buffer (10 mM Tris pH 6.8, 140 mM NaCl, 3 mM MgCl_2_, 0.5% NP-40, protease inhibitor cocktail (Sigma-Aldrich)) for 5 min on ice [19]. After clarification by centrifugation (1000×g, 5 min), the supernatant was retained as the cytosolic fraction. The pellet was washed with ice-cold TBN buffer (300 ul), and centrifuged at 1000×g for 5 min at 4°C. The nuclear pellet was further resuspended in 3 ml of S1 buffer (250 mM Sucrose, 10 mM MgCl_2_, protease inhibitor cocktail (Sigma-Aldrich)) and then it was layered over 3 ml of S3 buffer (880 mM Sucrose, 0.5 mM MgCl_2_, protease inhibitor cocktail (Sigma-Aldrich)) [26]. After centrifugation (2800×g, 10 min), the supernatant was discarded and the nuclear pellet resuspended in RIPA buffer (20 mM Tris-HCl pH 7.5, 50 mM NaCl, 5% Glycerol, 0.1% Triton X-100) containing protease inhibitor cocktail (Sigma-Aldrich) for 30 min on ice. Aliquots of the cytosolic and nuclear fractions were analyzed by SDS-PAGE and Western blot.

### SDS-PAGE and Western Blot Analysis

The cell lysate was prepared by incubating the cells with RIPA buffer (20 mM Tris-HCl pH 7.5, 50 mM NaCl, 5% Glycerol, 0.1% Triton X-100) containing protease inhibitor cocktail (Sigma-Aldrich) for 30 min on ice followed by sonication; then the lysate was mixed with protein sample buffer and boiled. The protein samples were subjected to 12% SDS-PAGE and then transferred to a PVDF membrane. The membranes were blocked in 5% milk in Tris-buffered saline containing 0.05% Tween 20 and probed with each of the following antibodies: GFP monoclonal antibody (abm), polyhedrin polyclonal antibody and CSFV-E2 monoclonal antibody (JENO Biotech). The membrane was then incubated with horseradish peroxidase-coupled anti-mouse IgG antibody (Cell Signaling), and the bound antibodies were detected using the enhanced chemiluminescence system (Merck Millipore) according to the manufacturer’s instructions.

### Fluorescence Spectrometer

Wild-type and EGFP fusion recombinant AcMNPV-infected cells were harvested by centrifugation at 1,000×g for 10 min, and the cell pellet was resuspended in 1 ml of PBS after washing with the same buffer. The lysate was prepared by incubating the cells with 900 µl of lysis buffer for 30 min on ice followed by sonication. Immediately, 100 µl of 1 M sodium carbonate was added, the resulting mixture was incubated at 37°C for up to 1 h, and then 2 ml PBS was added. Measurements were performed at room temperature in quartz cuvettes with a minimum test volume of 3 ml. The fluorescence intensity of the resulting mixture samples was measured using a K2™ fluorescence spectrometer (ISS Inc.) with an excitation filter of 450 nm and an emission filter of 510 nm. A minimum of three trials were conducted.

### Ethics Statement

Animal experimental procedures were approved by the Committee on the Use and Care on Animals (Permit Number: 100506-04, Institutional Animal Care and Use Committee of Choong-Ang Vaccine Laboratory, Daejeon, Korea) and performed in accordance with the institution guidelines.

### Immunization and Measurement of CSFV E2 in Antibody

Four guinea pigs were immunized with total cell extracts infected with either recombinant AcMNPV containing the E2 gene or wild-type AcMNPV. The prime immunization was performed using antigens that were mixed with Freund’s complete adjuvant (Sigma-Aldrich). Two weeks later, the guinea pigs were boosted with the same dose of antigen together with Freund’s incomplete adjuvant (Sigma-Aldrich). Blood samples were collected from the tail vein of each guinea pig at 2 weeks after the boost. The purified sera were analyzed for the presence of CSFV E2 antibody using the IDEXX CSFV Ab Test Kit (IDEXX, Me) according to the manufacturer’s instructions. The optical densities of the samples were measured at 450 nm using an ELISA microplate reader. The degree of CSFV E2 specific antibodies in the serum was calculated as follows: E2 blocking percentage = [(N-S)/N] × 100, where N is the mean OD_450_ of the negative control, and S is the mean OD_450_ of the test sample. The test samples were considered serologically positive when the blocking percentage was ≥40%.

## Results and Discussion

### Intracellular Localization and Assembly of the Partial Polyhedrin-fused Proteins

**Figure 1:**
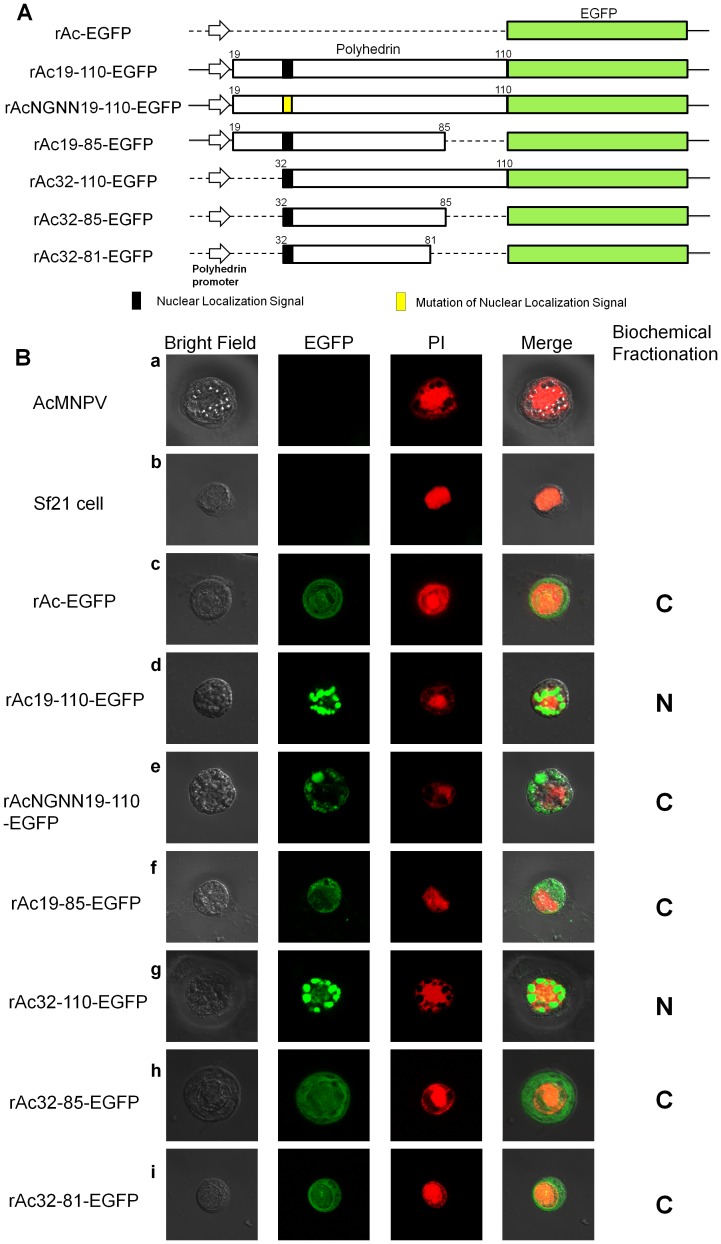
Schematic diagram and the intracellular distribution patterns of the fusion protein in Sf21 cells. (A) The polyhedrin fragments were genetically fused to the enhanced green fluorescent protein (EGFP) under the control of the polyhedrin promoter. Black shadowing corresponds to the basic residue cluster KRKK, and yellow shadowing corresponds to the mutative residue cluster NGNN. Subscript numbers refer to the polyhedrin amino acids fused to EGFP. (B) The fluorescence by EGFP was examined in Sf21 cells infected with viruses at 3 days post-infection. Cells were also stained to reveal the location of the nuclei (PI). Combining EGFP and PI staining revealed the intracellular location of EGFP (Merge). Protein localization was visualized using a confocal laser scanning microscope.

Several recombinant viruses were generated to express partial polyhedrin fused to the N-terminus of EGFP under the control of the AcMNPV polyhedrin promoter (Figure 1A). The fusion regions of polyhedrin were selected based on the previously reported minimal polyhedrin region for self-assembly (19 to 110 amino acids) and KRKK nuclear localization signal because we considered that the localization of recombinant proteins in the nucleus will be able to enhance their stabilities. The polyhedrin domain between amino acids 19 and 110 was used to express fusion proteins in the following forms: whole, N-, C- and with both terminal domains deleted. To determine which domains localize the fusion proteins to the nucleus, virus infected cells were observed using confocal laser scanning microscopy (Figure 1B). The result showed that the domain between amino acids 19 and 110 or 32 and 110 could mediate the nuclear localization of the fusion protein while other domains could not, even if the KRKK sequence is present as a minimal NLS. This suggests that the domain between 86 and 110 is required for the nuclear localization of polyhedrin. However, the nuclear localization of the fusion protein did not take place when the KRKK sequence was mutated to NGNN, similar to previous reports (Figure 1B) [17]. This indicates that the KRKK sequences are an essential requirement for the nuclear localization of polyhedron-fused recombinant protein, which is consistent with previous reports [17]. A nuclear localization signal is known to mediate the transport of nuclear proteins into the nucleus; therefore, its deletion disrupts the nuclear importation and enables fusion to a non-nuclear protein, which can be imported into the nucleus [27]. Our results also supported these facts. Although proteins do not contain an NLS, the transportation of proteins from the cytoplasm to the nucleus is possible either due to binding another polypeptide bearing an NLS or because it is smaller than approximately 60 kDa by diffusion [28], [29]. Partial polyhedrin-fused EGFP is smaller than 60 kDa, but it did not localize to the nucleus due to a mutation of the NLS (Figure 1B). This also indicates that the transportation of polyhedrin does not occur by diffusion, but instead it occurs by its NLS. The shortness of the fusion domain from polyhedrin amino acid 110 to 85 or 59 could not mediate nuclear localization. This suggested that some other essential region for nuclear localization is located between amino acids 85 and 110 of polyhedrin. Biochemical fractionation analysis showed also same result for the localization of recombinant protein by each virus (Figure S1). It was difficult to observe the formation of polyhedron-like particles in all of the virus-infected cells. However, the assembly of recombinant proteins was shown by rAc19-110-EGFP, rAc32-110-EGFP and rAcNGNN19-110-EGFP (Figure 1B). Although it was rare, the polyhedrin-like particles were produced by rAc19-110-EGFP (data not shown). Recombinant virus rAcNGNN19-110-EGFP did not completely produce those particles. This was a different result from that of the previously reported recombinant virus, which expresses β-galactosidase fused with the same NGNN-mutated partial polyhedrin [19]. The difference in fused genes between the two viruses may be one of reasons for this variation, but the results from rAc19-110-EGFP and rAcNGNN19-110-EGFP suggest that the assembly conditions, either within the nucleus or the cytoplasm, are also important.

### Enhanced Protein Production by the Polyhedrin Fusion Fragment

**Figure 2:**
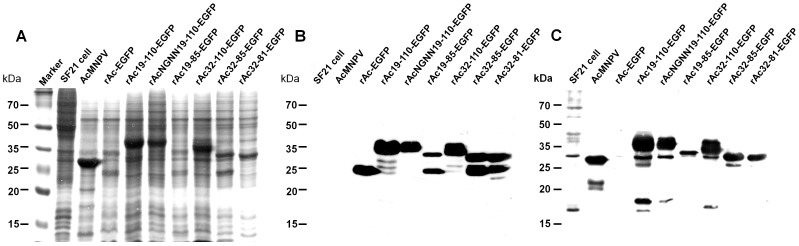
Comparative analysis of fusion protein production. Sf21 cells were infected at an MOI of 5 with each virus and harvested 4 days post-infection. Protein samples from the cells were analyzed by SDS-PAGE (A) and Western blot analysis with EGFP (B) and polyhedrin (C) antibodies.

To assess the effect of the polyhedrin fusion fragment on the level of recombinant protein production, a comparative analysis was performed at 48–96 h post-infection. The production of recombinant protein increased over time, but the differences between the viruses were not changed significantly depend on times (Figure 2, Figure S2). The native polyhedrin and the fusion proteins were identified on SDS-PAGE profiles according to their relative sizes, their absence in the Sf21 and the rAc-EGFP control lanes (Figure 2A). Additionally, the identification of each fusion protein was confirmed by Western blot analysis using anti-GFP and anti-polyhedrin (Figure 2B, C). The production level of EGFP appeared markedly increased by the partial polyhedrin fusion in most of the recombinant viruses, except for rAc19-85-EGFP. Although the exact comparison was difficult because of the different protein sizes, the production of the fused EGFP protein containing the region with amino acids 19 to 110 was remarkably similar to that of native polyhedrin (Figure 2A). This suggests that the production of recombinant protein by BEVS is able to reach the level of native polyhedrin. We considered that the enhanced production of recombinant protein is due to the nuclear localization of the produced protein by improving its stability and/or the existence of partial polyhedrin itself. The effect of nuclear localization was proved by rAcNGNN19-110-EGFP. The production of fusion proteins by rAcNGNN19-110-EGFP was still higher than by rAc-EGFP, but it was less than by rAc19-110-EGFP (Figure 2A). This indicates that the partial polyhedrin fusion is important for not only the production of foreign proteins itself but also for the nuclear localization of protein. Our results did not indicate whether the assembly of protein or the nucleus condition is more important for the enhanced production of foreign protein. The enhanced production of foreign protein by rAc19-110-EGFP and rAc32-110-EGFP, showing the most stable assembly of the protein (Figure 1B), suggests that the assembly of a foreign protein also influences the stability of the foreign protein. The importance of the nuclear localization of foreign proteins for enhanced stability was also reported in plant cells [30]. Nuclear localization of foreign protein in tobacco cells enhanced its accumulation in the cytosol due to the enhanced stability of the foreign protein, as verified by enzymatic assays and RNA levels. The enhanced stability and expression of foreign proteins by supramolecular self-assembly was also reported using human ferritin heavy chain (FTN-H) [31], [32]. The fusion expression of FTN-H increased the production and solubility of foreign protein. The enhanced expression and solubility of foreign protein by a polyhedrin fusion was also reported in *Escherichia coli* [33], [34]. However, these studies examined each of these effects (i.e., nuclear localization or assembly of foreign proteins for enhanced production) separately. Therefore, the fusion expression of partial polyhedrin, which could be expected to display both of these effects, may be a better approach for producing foreign proteins.

The effect of partial polyhedrin itself, and not just assembly or nuclear localization, was also seen in other recombinant viruses, which had variable polyhedrin fusion regions (Figure 2A). These observations suggest that the partial polyhedrin is able to enhance the stability of protein by changing the protein structure and/or preventing proteolytic degradation. The importance of the N-terminal residue of the protein during degradation is suggested by the N-end rule pathway [35]-[37]. Although we could not be sure that a change in the N-terminal residue by a partial polyhedrin fusion decreases proteolysis, the presence of partial polyhedrin might influence its susceptibility to proteolytic degradation. Further studies are needed to determine whether the N-terminal residue of the fused polyhedrin is related to proteolysis, and this may increase the effect of the partial polyhedrin fusion. It was not shown directly that the change in protein structure could influence its stability, but similar results were observed. The intact EGFP, including polyhedrin fused-EGFP, was also detected in rAc19-85-EGFP and rAc32-85-EGFP infected cells (Figure 2B). Detection of EGFP indicated that the degradation and/or cleavage of fused polyhedrin from fusion protein. As amino acids 82 to 85 are known to form β-sheets in polyhedrin [20], rAc32-81-EGFP was generated to investigate the effect of this region. Deletion of amino acids 82 to 85 decreased the production and cleavage of recombinant protein (Figure 2B). Degraded or cleaved polyhedrin was not observed, and the reasons for where and why it cleaved were not clearly determined in this study; however, these results suggested that the polyhedrin fusion may mediate the change in protein structure, and this may be the reason for the deletion of polyhedrin. Additionally, the increased production of recombinant protein by rAc32-85-EGFP showed the usefulness of partial polyhedrin for the enhanced and intact production of recombinant protein. Further studies are required to define the most suitable region for these fusions.

**Figure 3:**
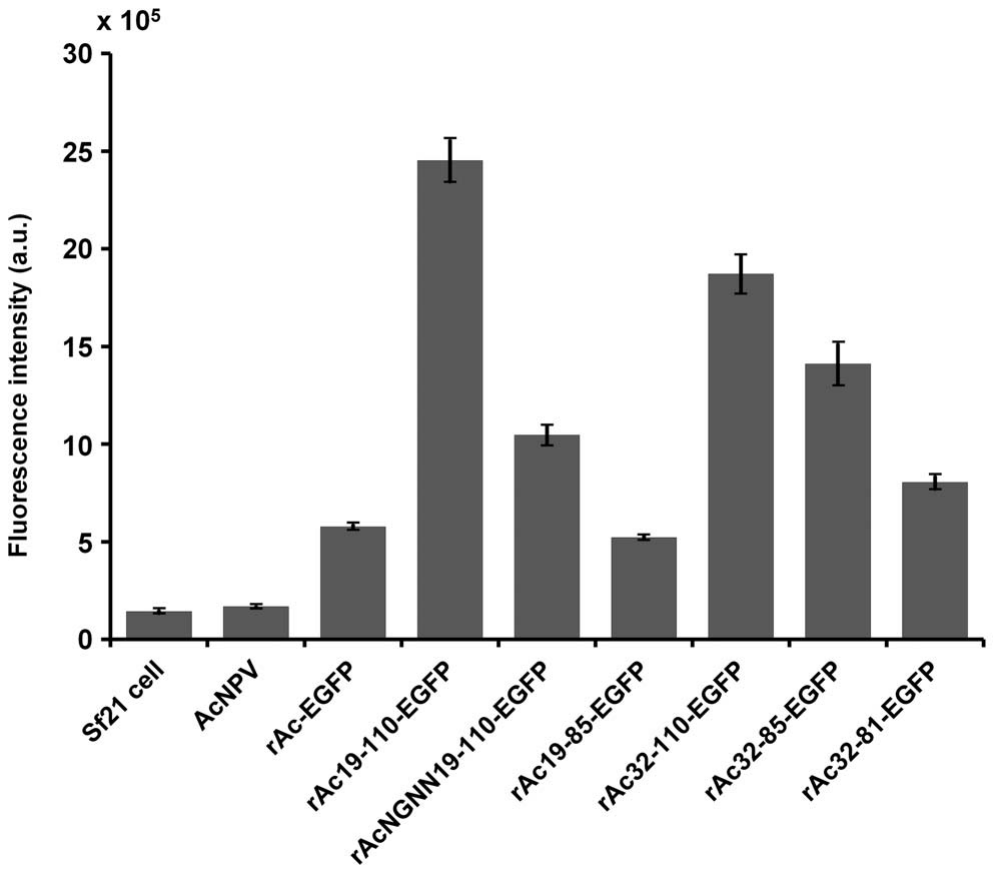
Fluorescence intensity of EGFP. Sf21 cells were infected at an MOI of 5 with each virus and harvested 3 days post-infection. The fluorescence intensity of the cell extracts was measured using a fluorescence spectrometer (B). The bars indicate the mean ± SE (*n* = 3).

The activity of polyhedron-fused EGFP was quantified using a fluorescence spectrophotometer (Figure 3). The results were similar to those obtained by SDS-PAGE analysis. The recombinant viruses rAc19-110-EGFP and rAc32-110-EGFP showed higher fluorescence levels than the others. This result clearly suggests that the localization of processed recombinant protein in the nucleus is better than in the cytosol.

### Expression of CSFV E2

**Figure 4:**
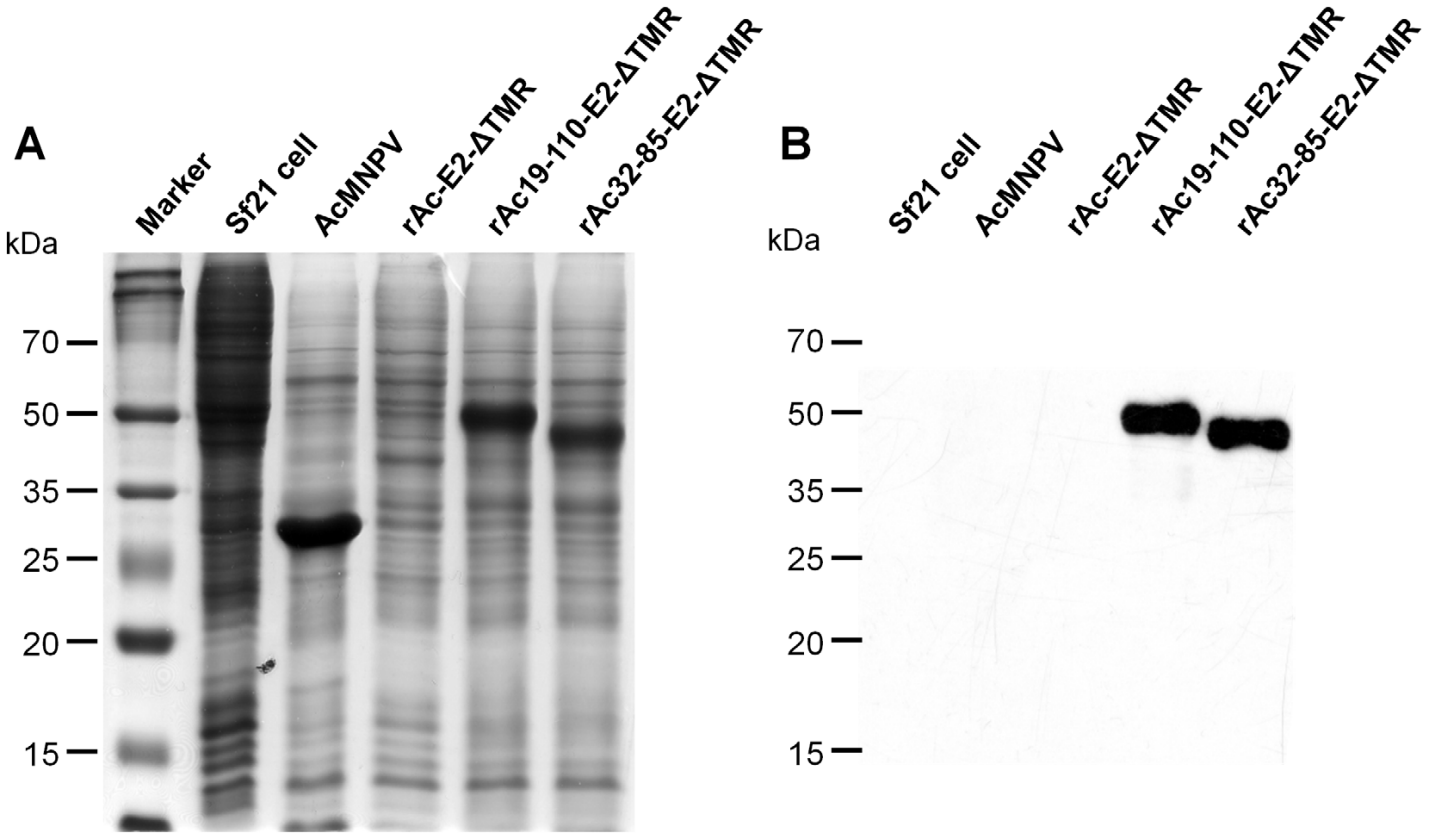
Expression of CSFV E2 protein fused with partial polyhedrin. Sf21 cells were infected at an MOI of 5 with each virus and harvested at 4 days post-infection. Protein samples were analyzed by SDS-PAGE (A) and Western blot analysis with E2 monoclonal antibody (B).

To investigate the utility of partial polyhedrin, a CSFV E2 was expressed both as fusion with polyhedrin residues 19 to 110 and residues 32 to 85. E2 is the major CSFV immunogen and has been used to develop subunit vaccines [38]. Production of E2 by rAc-E2-ΔTMR was not observed at the protein level by SDS-PAGE and Western blot analyses at 48-96h post-infection (Figure 4, Figure S3). The existence of the E2 gene in rAc-E2-ΔTMR was confirmed by PCR analysis (Figure S4). The expression of E2 by rAc-E2-ΔTMR was identified on the mRNA level by RT-PCR (Figure S5). In contrast, when expressed as fusion proteins with polyhedrin fragments, the production of E2 was very high in both rAc19-110-E2-ΔTMR and rAc32-85-E2-ΔTMR (Figure 4). To confirm the virus infection, the yield of virus was compared to each virus derived from infected cells (Figure S6). The results showed no difference in the viral growth for each virus, suggesting that a CSFV E2 could be hyper-expressed by using a partial polyhedrin fusion. Meanwhile, the cleavage of a fused-polyhedrin residue from the recombinant protein by rAc32-85-E2-ΔTMR was not observed (Figure 4). This demonstrated that the cleavage and/or degradation of the polyhedrin residue from the fusion protein may depend on the structure of protein, and this may vary according to the fused protein.

**Figure 5:**
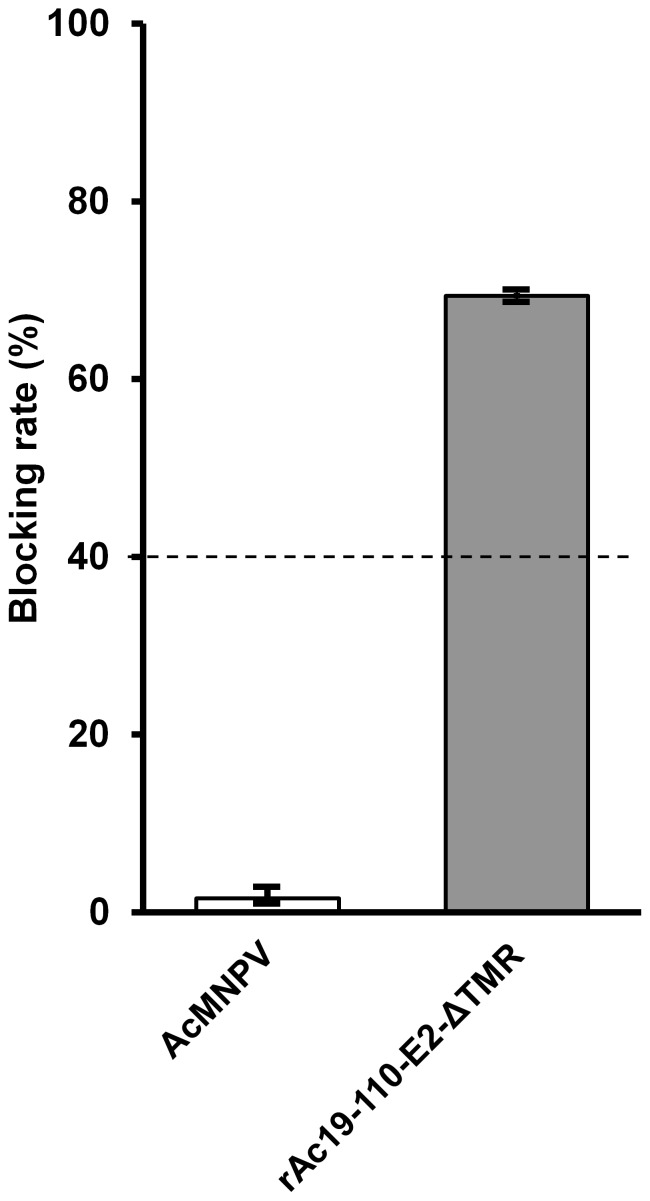
Blocking rate of E2 antibodies induced in the sera of guinea pigs immunized with rAc-19-110-E2- ΔTMR. Four guinea pigs were immunized with total cell extracts infected with rAc19-110-E2-ΔTMR and wild type AcMNPV for 4 weeks. The levels of E2-specific antibodies in the sera were measured using the IDEXX CSFV Ab Test Kit. The optical densities of the samples were measured at 450 nm using an ELISA microplate reader. The test samples were considered serologically positive when the blocking percentage was ≥40% (dotted line). The blocking percentages are expressed as the mean ± SE (n = 4).

The immunization efficacy of recombinant E2 protein was preliminarily tested in guinea pigs. Four guinea pigs were immunized with total cell extracts infected by rAc19-110-E2-ΔTMR. The E2-specific humoral response was assessed using a commercial ELISA kit (IDEXX CSFV Ab Test, IDEXX Co.). The blocking rate was 69.4% by the sera of the E2-immunized guinea pigs, but the control group blocking rate remained lower than 2% (Figure 5). This result suggested that the antibodies induced by the polyhedrin fragment-fused E2 can provide adequate protection against CSFV. However, the most convincing evidence of the immunogenicity of this antigen should be achieved from vaccination experiments with swine.

In conclusion, this study showed the possibility for the enhanced production of foreign protein using fusion expressions with various polyhedrin fragments. The results suggest that the reason for enhanced production may be due to either nuclear localization or the assembly of protein and partial polyhedrin itself. Additionally, the substantial production of CSFV E2 strongly proved the usefulness of this fusion expression system. Further studies that explore the expression of other foreign genes will provide additional information on this fusion expression system.

## Supporting Information

Figure S1 Intracellular localization of fusion protein production. Sf21 cells were infected at an MOI of 5 with each virus and harvested at 3 days post-infection. Cells were separated into nuclear (A) and cytosolic (B) fractions by detergent-based procedure. Fractions were analyzed by SDS-PAGE (left panel) and Western blot analysis with EGFP antibody (right panel).
(TIF)

Figure S2 Comparative analysis of fusion protein production. Sf21 cells were infected at an MOI of 5 with each virus and harvested at 2 (A) and 3 (B) days post-infection. Protein samples from the cells were analyzed by SDS-PAGE (left panel) and Western blot analysis with EGFP antibody (right panel).
(TIF)

Figure S3 Expression of CSFV E2 protein fused with partial polyhedrin. Sf21 cells were infected at an MOI of 5 with each virus and harvested at 4 days post-infection. Protein samples were analyzed by SDS-PAGE (A) and Western blot analysis with E2 monoclonal antibody (B).
(TIF)

Figure S4 PCR analysis for the presence of CSFV E2-ΔTMR gene in viral DNA. PCR amplification was performed for viral DNA isolated from Sf21 cells infected with the AcMNPV or rAc-E2-ΔTMR. The CSFV E2 gene specific primers were used to identify it.
(TIF)

Figure S5 Comparison of viral growth between AcMNPV and rAc-E2-ΔTMR. Sf21 cells were infected with AcMNPV or rAc-E2-ΔTMR at 5 MOI. The cell culture supernatants were harvested and titrated by TCID_50_ endpoint dilution assays for the presence of infectious budded virus. The results represent the average titers derived from three independent assays. The error bars represent standard errors.
(TIF)

Figure S6 RT-PCR analysis of the expression of the CSV E2-ΔTMR gene by the recombinant virus. The Sf21 cells were infected with AcMNPV or rAc-E2- ΔTMR at 5 MOI. Total RNA from infected cells was collected and subjected to reverse transcription-PCR, and the products were analyzed by electrophoresis on 1% agarose gels.
(TIF)
