# Supplementary figures and images for: Hyper-Enhanced Production of Foreign Recombinant Protein by Fusion with the Partial Polyhedrin of Nucleopolyhedrovirus
Source: PLoS One. 2013 Apr 9;8(4):e60835. doi: 10.1371/journal.pone.0060835 (PMC3621880; doi:10.1371/journal.pone.0060835)

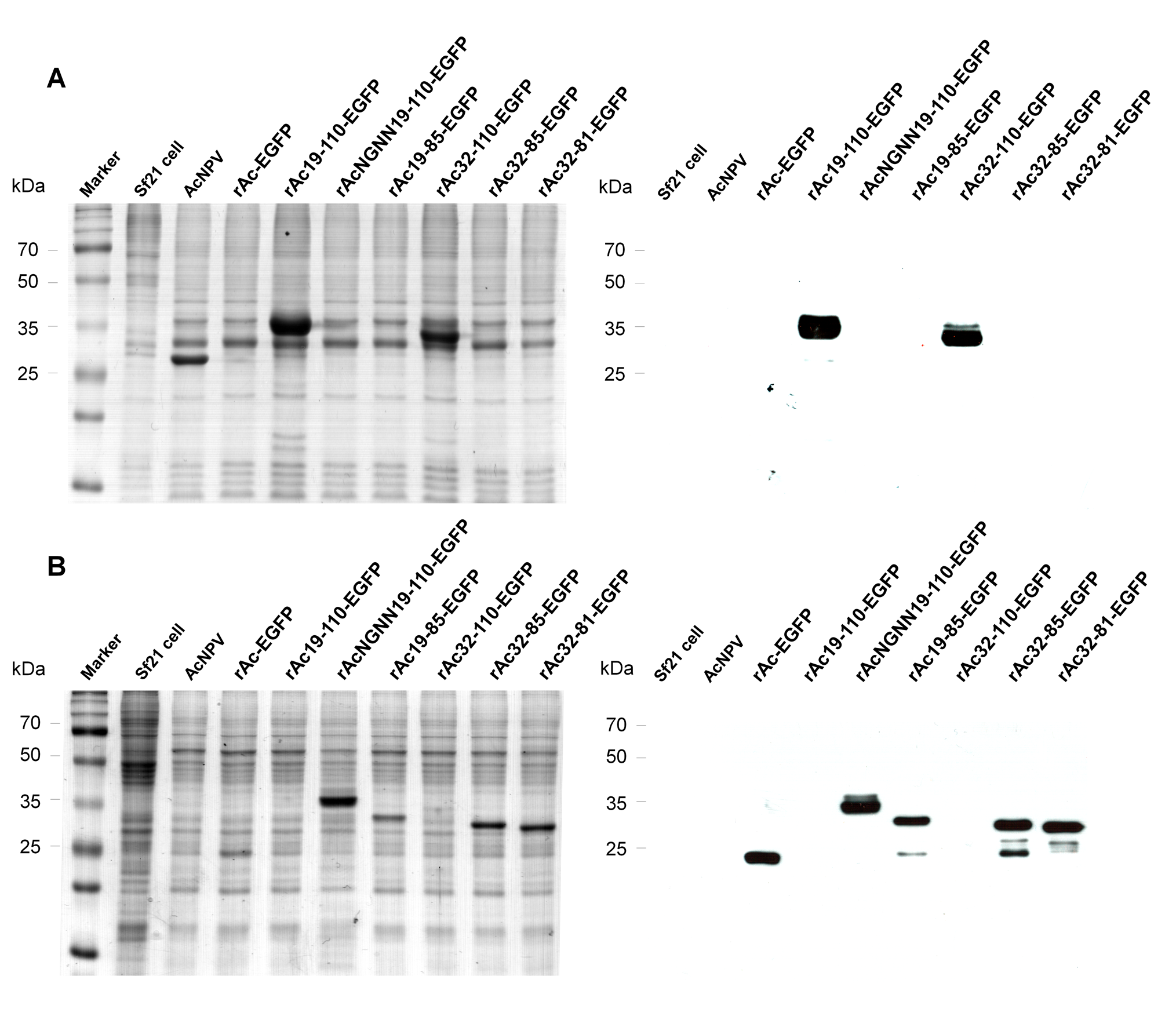

Supplement: Figure S1 — Intracellular localization of fusion protein production. Sf21 cells were infected at an MOI of 5 with each virus and harvested at 3 days post-infection. Cells were separated into nuclear (A) and cytosolic (B) fractions by detergent-based procedure. Fractions were analyzed by SDS-PAGE (left panel) and Western blot analysis with EGFP antibody (right panel). (TIF) [file pone.0060835.s001.tif]

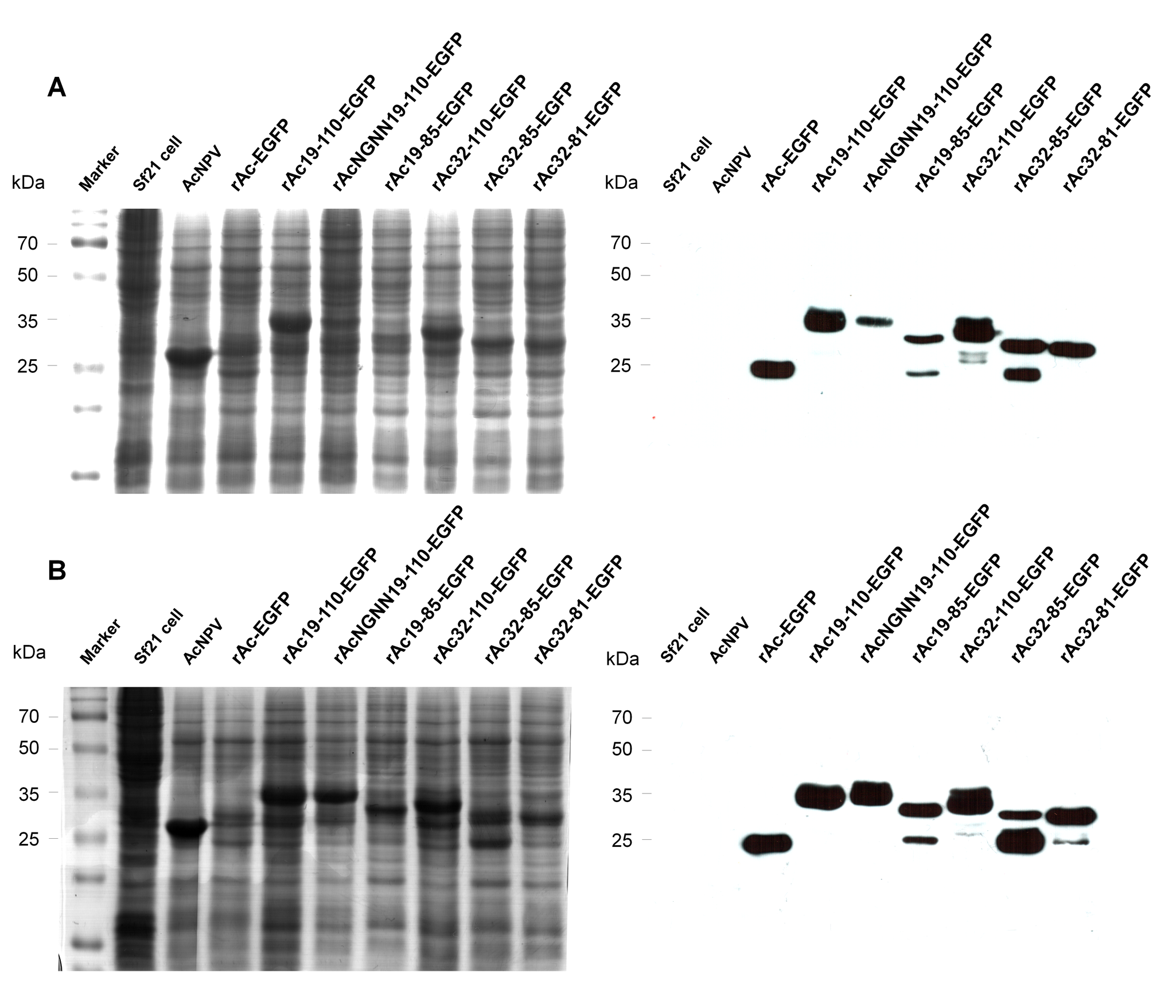

Supplement: Figure S2 — Comparative analysis of fusion protein production. Sf21 cells were infected at an MOI of 5 with each virus and harvested at 2 (A) and 3 (B) days post-infection. Protein samples from the cells were analyzed by SDS-PAGE (left panel) and Western blot analysis with EGFP antibody (right panel). (TIF) [file pone.0060835.s002.tif]

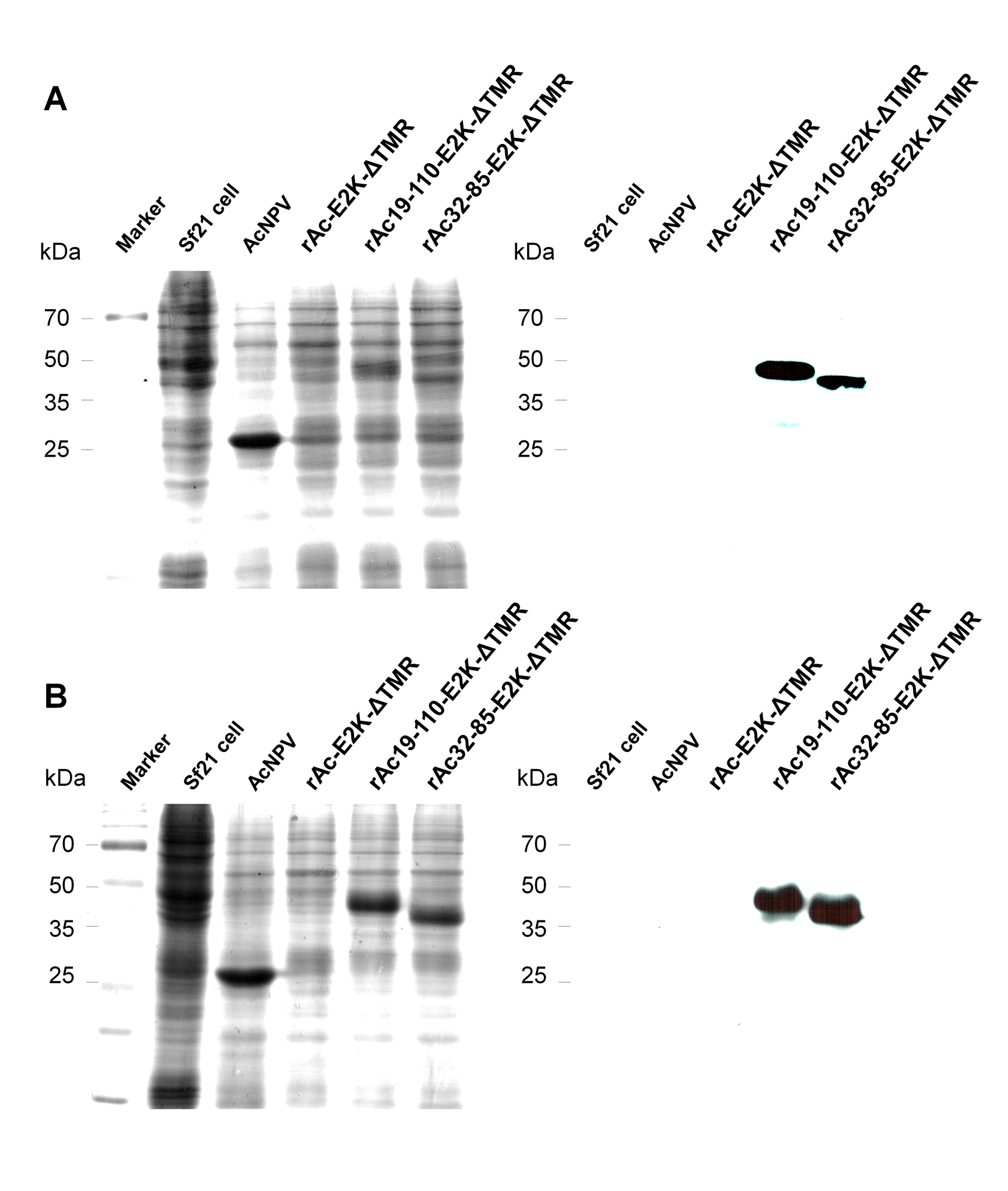

Supplement: Figure S3 — Expression of CSFV E2 protein fused with partial polyhedrin. Sf21 cells were infected at an MOI of 5 with each virus and harvested at 4 days post-infection. Protein samples were analyzed by SDS-PAGE (A) and Western blot analysis with E2 monoclonal antibody (B). (TIF) [file pone.0060835.s003.tif]

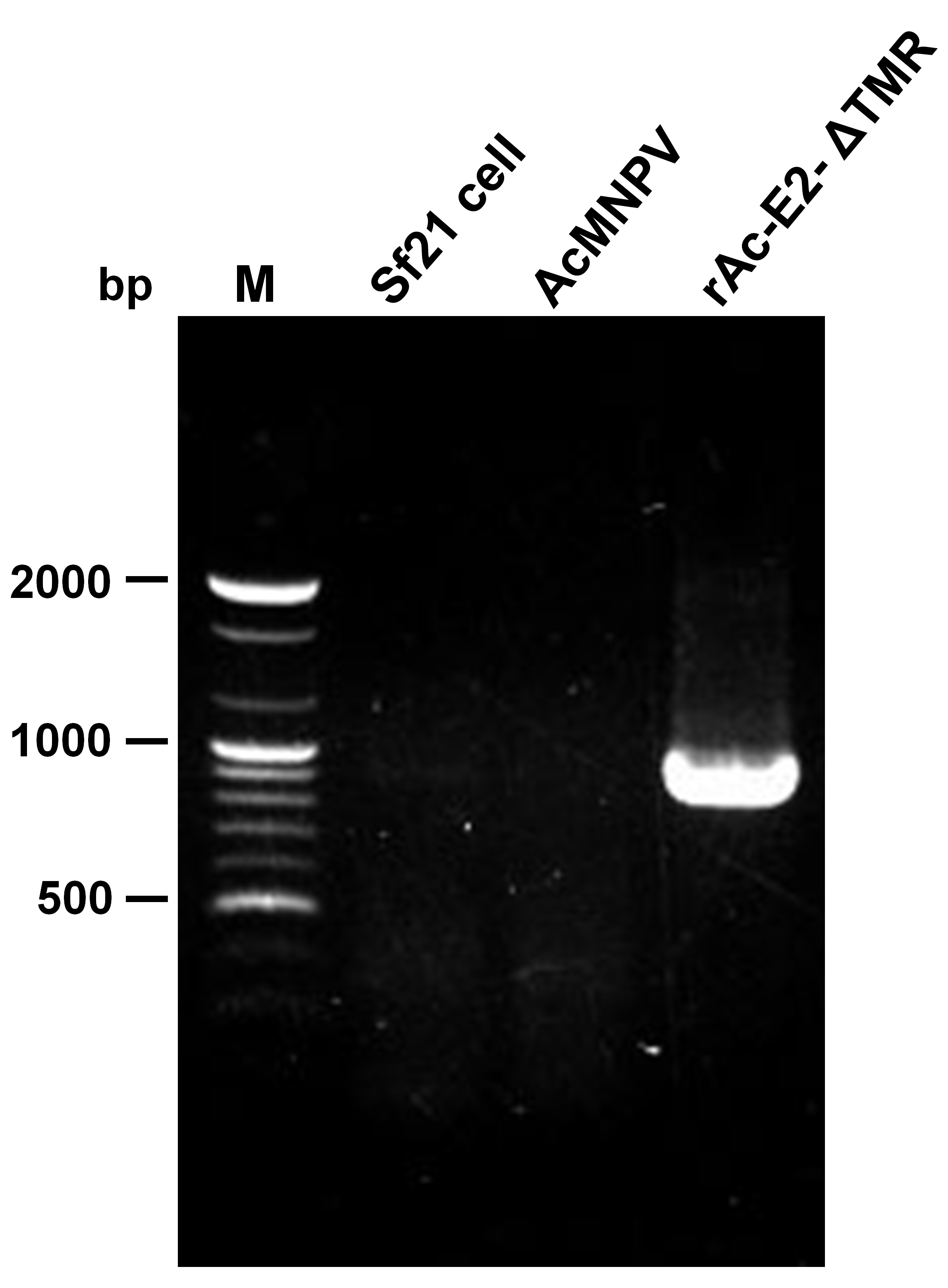

Supplement: Figure S4 — PCR analysis for the presence of CSFV E2-ΔTMR gene in viral DNA. PCR amplification was performed for viral DNA isolated from Sf21 cells infected with the AcMNPV or rAc-E2-ΔTMR. The CSFV E2 gene specific primers were used to identify it. (TIF) [file pone.0060835.s004.tif]

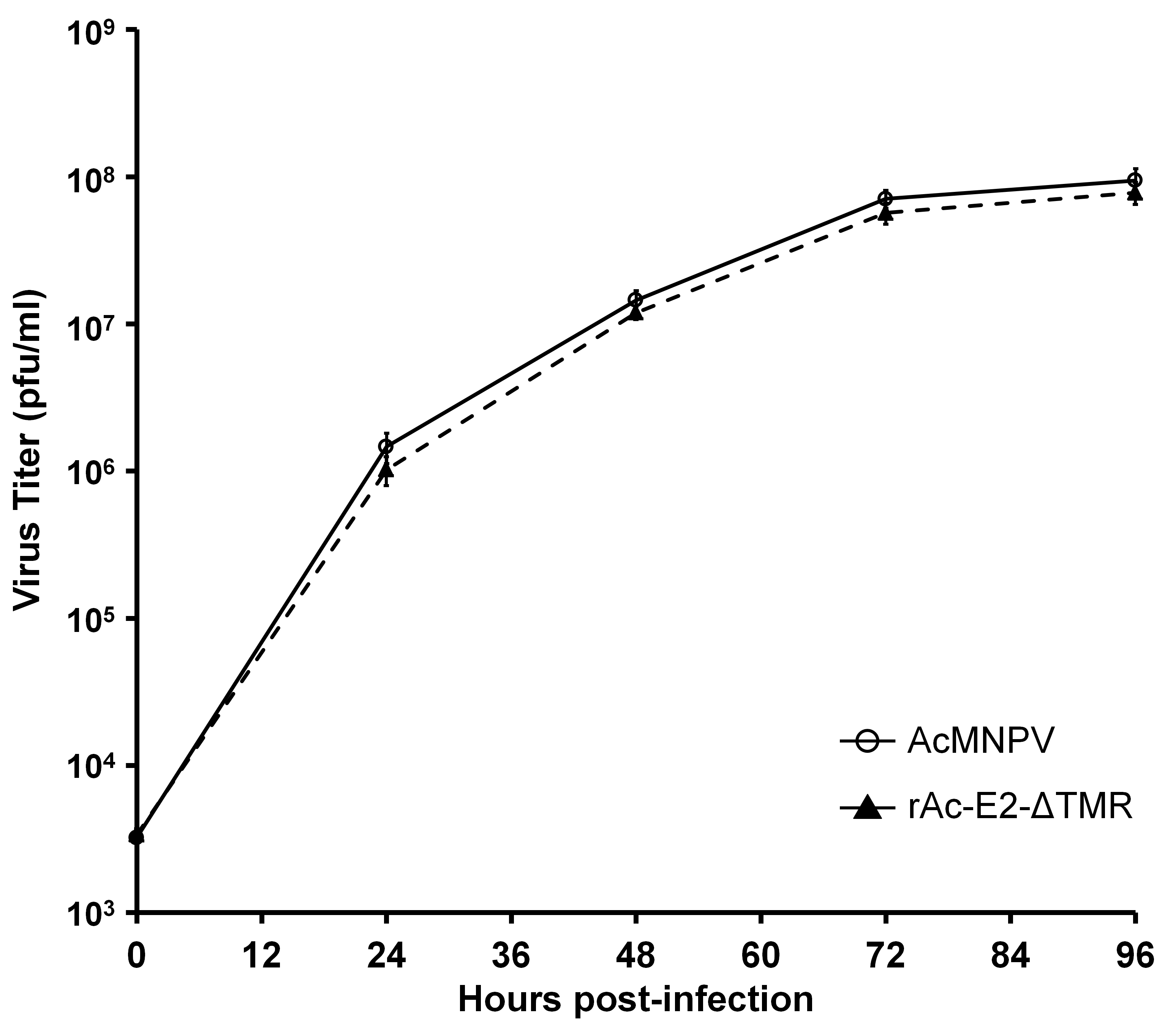

Supplement: Figure S5 — Comparison of viral growth between AcMNPV and rAc-E2-ΔTMR. Sf21 cells were infected with AcMNPV or rAc-E2-ΔTMR at 5 MOI. The cell culture supernatants were harvested and titrated by TCID50 endpoint dilution assays for the presence of infectious budded virus. The results represent the average titers derived from three independent assays. The error bars represent standard errors. (TIF) [file pone.0060835.s005.tif]

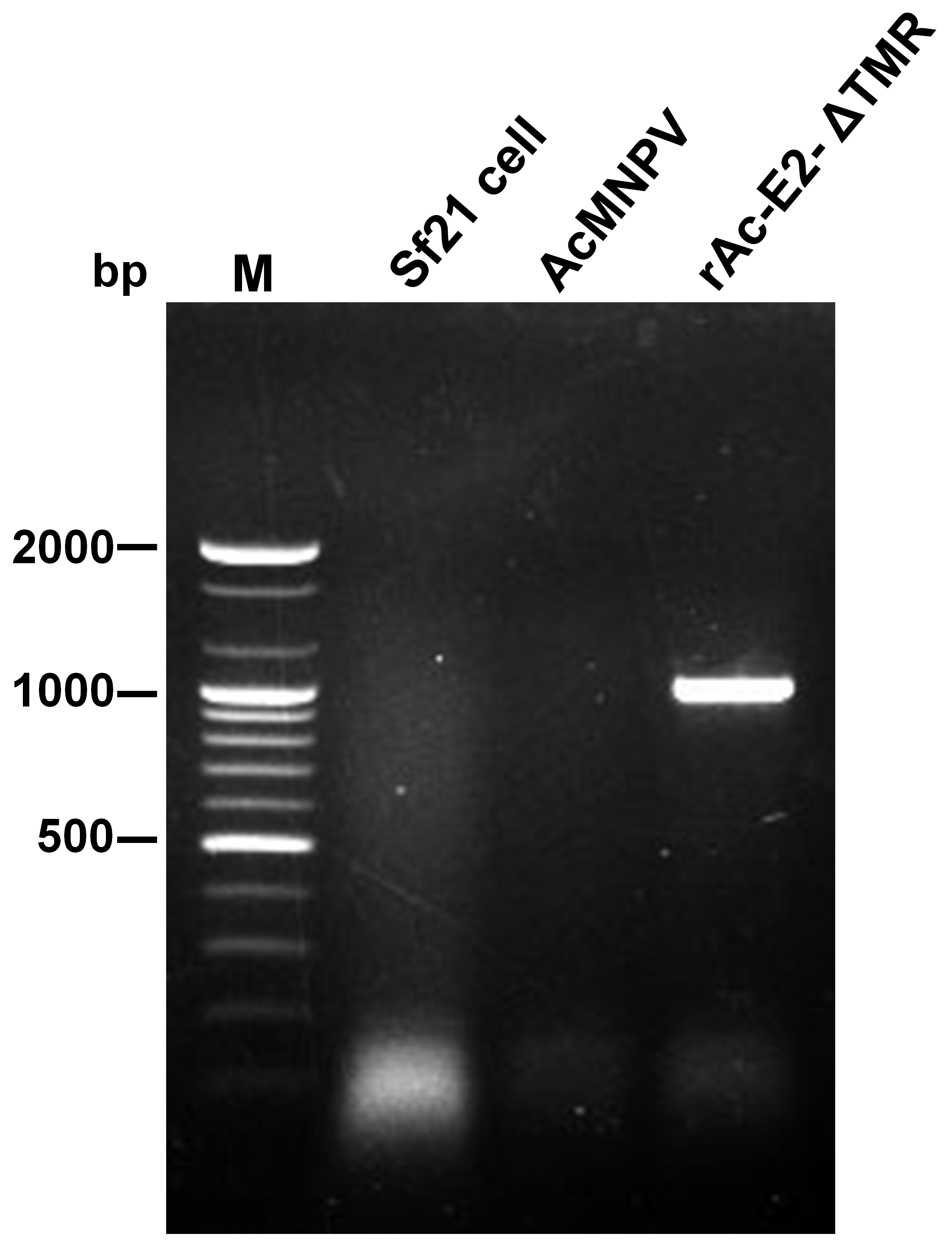

Supplement: Figure S6 — RT-PCR analysis of the expression of the CSV E2-ΔTMR gene by the recombinant virus. The Sf21 cells were infected with AcMNPV or rAc-E2- ΔTMR at 5 MOI. Total RNA from infected cells was collected and subjected to reverse transcription-PCR, and the products were analyzed by electrophoresis on 1% agarose gels. (TIF) [file pone.0060835.s006.tif]
